# Regional Anesthesia Modalities for Spine Surgery: A Narrative Review

**DOI:** 10.1007/s11916-026-01579-0

**Published:** 2026-11-14

**Authors:** Elizabeth Kim, Caitlin Krol, Brian H. Park, Engy T. Said, Rodney A. Gabriel

**Affiliations:** 1 Division of Regional Anesthesia and Acute Pain, Department of Anesthesiology, University of California, San Diego, San Diego, CA USA; 2 Department of Anesthesiology and Perioperative Care, University of California, Irvine, CA Irvine, USA

**Keywords:** Fascial plane blocks, Spine surgery, Regional anesthesia, Erector spinae plane block

## Abstract

**Purpose of Review:**

This narrative review examines the evidence supporting regional and neuraxial analgesic techniques for spine surgery, with particular emphasis on interfascial plane blocks, peripheral regional techniques, and neuraxial analgesia.

**Recent Findings:**

Randomized trials and meta-analyses suggest that regional analgesic techniques can reduce postoperative pain and opioid consumption following spine surgery; however, the strength and consistency of these findings vary considerably. Reduction in postoperative nausea and vomiting is among the most consistent benefits. Neuraxial techniques may provide more profound and prolonged analgesia but are accompanied by the potential for more consequential adverse effects.

**Summary:**

Current evidence does not clearly establish the superiority of a single regional analgesic technique for spine surgery. Clinicians may benefit from developing technical proficiency in a limited repertoire of regional techniques and applying them at an anatomically appropriate level for the planned surgery.

## Introduction

With longer life expectancies and aging of the general population, the prevalence of degenerative spinal diseases and the number of associated surgical procedures performed have increased [1, 2]. The annual frequency of lower back pain is approximately 10–15% among healthy adults [3]. While acute intervertebral disc herniation often resolves with conservative management [3], nearly 300,000 lumbar discectomies are performed annually in the United States [4]. Complex spine surgery, defined as “thoracolumbar spine surgery with instrumentation, laminectomy at three or more levels, or scoliosis surgery,” are often expected to be accompanied by significant postoperative pain [5]. Postoperative surgical pain is often complex, with contributing factors including incisional pain; nociceptive, neuropathic, or inflammatory pain originating from deeper structures such as the ligaments, muscles, intervertebral discs, facet joints, and damaged nerve roots; pain from extensive surgical dissection and muscle retraction; and prolonged operating time [6–10].

Inadequate postoperative pain management is associated with delayed mobilization and functional recovery, development of chronic pain and opioid dependence, lung atelectasis, prolonged hospital stay, and decreased patient satisfaction and quality of life [3, 4, 9–12]. Conversely, adequate pain control following surgery is associated with earlier mobilization and discharge [2, 5]. Traditionally, opioids have been the mainstay of postoperative pain management. Recognizing the limitations and risk of adverse effects such as nausea, vomiting, sedation, ileus, pruritus, dependence, and respiratory depression [3, 9–11, 13], alternative non-opioid treatments have been sought. Various multimodal strategies include acetaminophen/paracetamol, non-steroidal anti-inflammatory drugs (NSAIDs), epidural steroid injections, and regional anesthetic procedures [3, 5, 7, 13, 14]. Intraoperatively, infusions of intravenous (IV) ketamine, lidocaine, or dexmedetomidine, and administration of IV methadone have been studied [5, 12], with recommendations supporting the use of low-dose ketamine (0.2-0.5 mg/kg bolus and 2 mg/kg/min continuous infusion) [5]. Even with the advent of enhanced recovery after surgery (ERAS) protocols advocating early mobilization and recovery, reduction of opioid-related adverse effects, and reduced PONV, the role of opioid-free analgesic regimens remains controversial [9].

With regional anesthetic techniques, anesthesiologists may provide more targeted pain relief while decreasing the need for systemic analgesia [2, 3, 10]. This narrative review seeks to provide a summary of various regional anesthetic techniques used for spine surgery (Table 1).

**Table 1:** Overview of regional and neuraxial techniques for analgesia for spine surgery

Technique	Primary target/ mechanism	Common spine procedures	Advantages	Limitations	Quality of evidence
Erector Spinae Plane (ESP)	Fascial plane deep to erector spinae muscle. Variable spread to dorsal and ventral rami with possible spread to intercostal spaces (laterally) or paravertebral and epidural spaces (medially).	Cervical, thoracic, lumbar decompression and fusion; pediatric spinal fusion	Technically simple; versatile across spinal levels; extensive craniocaudal spread; reduced postoperative opioid consumption, pain scores, rescue analgesia, and PONV; favorable safety profile	Variable injectate spread; modest opioid reduction; potential brachial plexus or phrenic nerve involvement with cervical blocks; optimal injection level remains uncertain	Largest evidence base among peripheral blocks with numerous RCTs and multiple meta-analyses
Thoracolumbar Interfascial Plane (TLIP)	Dorsal rami of thoracolumbar nerves between multifidus-longissimus (classical) or longissimus-iliocostalis (modified).	Posterior lumbar discectomy, decompression, laminectomy, lumbar fusion	Sensory-selective block; excellent posterior lumbar coverage; modified approach technically easier; consistently superior to no block; reduction in postoperative opioid consumption	Limited to lumbar surgery; conflicting comparative evidence versus ESP; few large RCTs	Multiple prospective trials and meta-analyses demonstrating benefit over control
Retrolaminar Block (RLB)	Local anesthetic deposited superficial to lamina with spread toward paravertebral space targeting the paraspinal nerves.	Lumbar decompression, lumbar discectomy	Easy sonoanatomy; less motor blockade than paravertebral block; effective early postoperative analgesia	Less extensive spread than ESP; fewer clinical studies; less robust analgesia in head-to-head comparisons	Small RCTs, cadaveric and MRI studies
Inter-semispinal Fascial Plane Block (ISPB)	Dorsal cervical rami within fascial plane between semispinalis cervicis and semispinalis capitis muscles.	Posterior cervical decompression, cervical fusion, suboccipital procedures	Preserves ventral roots, brachial plexus, phrenic nerve, MEPs and SSEPs; technically straightforward; promising opioid reduction	Very limited clinical literature; mostly single-center studies; no large comparative trials	Two RCTs, cadaveric studies, pediatric case series
Epidural	Epidural spread surrounding nerve roots and dorsal root ganglia via catheter or single injection.	Complex lumbar fusion, multilevel open spine surgery	Excellent analgesia; strong opioid-sparing effect; guideline-supported; continuous catheter option	Motor weakness, urinary retention, hypotension, delayed neurologic assessment, catheter failure, hematoma risk	Multiple RCTs, meta-analyses, ERAS and PROSPECT recommendations
Intrathecal Morphine (ITM)	µ-opioid receptor activation within dorsal horn of spinal cord.	Lumbar fusion, deformity surgery, open spine procedures	Potent prolonged analgesia (up to 24 h); substantial opioid reduction; technically simple	Pruritus, urinary retention, respiratory depression, sedation; limited duration; monitoring required	Multiple RCTs and meta-analyses; mixed guideline recommendations

## Human and Animal Rights

All reported studies/experiments with human or animal subjects performed by the authors have been previously published and complied with all applicable ethical standards (including the Helsinki declaration and its amendments, institutional/national research committee standards, and international/national/institutional guidelines).

## Methods

Google Scholar and PubMed were used to search for relevant articles. For PubMed, searches were filtered by limiting to the specific article types: “Clinical Trial”, “Meta-Analysis”, “Systematic Review”, “Review”, and “Randomized Controlled Trial”. To search for literature related to erector spinae plane blocks, the following search terms were used: (“erector spinae plane block” or “erector spinae block”) and “spine surgery”. For thoracolumbar interfascial plane (TLIP) blocks, the following search terms were used: (“TLIP” or “thoracolumbar interfascial plane block”) and “spine surgery”. For retrolaminar block, we used “retrolaminar block” and “spine surgery”. For inter-semispinal fascial plane block (ISBP), we used: (“cervical cervicis plane block” or “inter-spinal fascial plane block”) and “spine surgery”. For epidural analgesia, we used “epidural analgesia” and “spine surgery”. For intrathecal morphine, we used “intrathecal morphine” and “spine surgery”.

## Erector Spinae Plane Blocks

### Mechanism of Action

The erector spinae plane (ESP) block was first described by Forero et al. in 2016 for ipsilateral thoracic analgesia [15]. The ESP block targets both the ventral and dorsal branches of the spinal nerves that live in the fascial plane deep to the erector spinae muscle at the level of the transverse process [7, 11, 16–18]. The injectate spreads in a craniocaudal distribution along several vertebral levels. At the level of the cervical spine, spread of local anesthetic into the prevertebral space may also block the brachial plexus and phrenic nerve [11, 18, 19]. Other areas that may receive distributions of local anesthetic include the intercostal spaces more laterally at the level of the thoracic spine [12, 18] and the paravertebral and epidural spaces more medially [8, 17, 18].

### Technique

The muscles of the erector spinae include the spinalis, longissimus, and iliocostalis, which extend on either side of the spine from the skull cranially to the pelvis caudally and to the ribs laterally [12]. Under ultrasound guidance, the block needle is inserted 2–3 cm lateral to the target spinous process and is advanced until contact is made with the desired transverse process. Local anesthetic is deposited in the fascial plane deep to the erector spinae muscle and superficial to the tip of the transverse process [16, 18]. While the block may be performed bilaterally and at multiple levels, the risk of toxicity should be considered when large volumes of local anesthetic are injected, in pediatric patients, and/or in patients with impaired hepatic function or slower clearance [8].

### Outcomes

Of the peripheral nerve block techniques discussed here, the ESP block has perhaps the longest publication history. The 2021 PROSPECT (PROcedure SPECific postoperative pain managemenT) Working Group guidelines for pain management following complex spine surgery determined that “data were insufficient to recommend the use of” lateral TLIP (see below) or ESP blocks “although results from recent publications are promising” [5]. Since then, several randomized controlled trials (RCT) and meta-analyses have been published, including a recent meta-analysis by Dost et al. [20] Their analysis of 60 RCTs - encompassing a large spectrum of vertebral surgeries - found that usage of ESP blocks resulted in modest reductions in 24-hour opioid consumption, improved pain scores at rest and with movement in the first 48 h, and decreased rescue analgesic requirements and postoperative nausea/vomiting (PONV). While the results regarding opioid consumption and pain scores were supported by low-certainty evidence and substantial unexplained heterogeneity, the significant reduction in PONV incidence was supported by high-certainty evidence and no between-study heterogeneity, leading the authors to conclude that the ESP block’s “clinical impact remains highly context dependent and likely influenced by unmeasured procedural and methodological factors”. Although no block-related complications were reported, the studies included were also not powered to detect adverse events. Rather, the risk of motor weakness due to spread to the brachial plexus and diaphragmatic paralysis have been discussed more in literature comparing ESP blocks to other regional techniques [18, 19]. Dost et al. were unable to comment on chronic postsurgical pain outcomes due to limited and heterogenous data [20].

ESP blocks have also been studied in the pediatric population, with a meta-analysis by Al-Naseem et al. including five studies of adolescent patients undergoing spinal fusion. This meta-analysis reported decreased intraoperative and postoperative opioid consumption, decreased pain scores up to 24 h postoperatively, decreased rescue analgesia requirement, and increased time to first rescue analgesia in the treatment population [8]. Time to ambulation, length of hospital stay, incidence of opioid-related side effects, and percentage change in motor evoked potentials (despite one case report documenting unilateral minimal reduction in MEPs following ESPB) were comparable with control.

## Thoracolumbar Interfascial Plane Block

### Mechanism

First described by Han et al. in 2015, the TLIP block targets the dorsal rami of the thoracolumbar nerves that travel through the paraspinal muscles and that provide sensory innervation to the back [2, 6, 13, 21–23]. In comparison to Hand et al.’s classical approach (cTLIP) that targets the fascial plane between the multifidus and longissmus muscles, the modified approach (mTLIP) – described in 2017 by Ahiskalioglu et al. – targets the plane between the longissimus and iliocostalis muscles [24, 25].

### Technique

With the patient in the prone position, the spinous process and interspinal muscles (from medial to lateral: multifidus, longissimus, and iliocostalis) are identified using ultrasound guidance [22, 23]. In the classical approach, the local anesthetic is deposited into fascial plane between the multifidus and longissimus muscles at the level of the third lumbar vertebrae using a lateral-to-medial approach. In the modified approach, the needle is advanced from medially to laterally through the iliocostalis muscle to deposit local anesthetic solution into the fascial plane between the longissimus and iliocostalis muscles [9, 22, 23].

### Outcomes

The efficacy of the TLIP block compared to control has been shown in several prospective studies and meta-analyses [2, 9, 11, 13]. More current research has focused more on the efficacy of the TLIP block as compared to other regional techniques as well as the difference between classical and modified approaches to TLIP.

Several studies have compared the efficacy of TLIP and ESP blocks in spine surgery. A 2023 frequentist network meta-analysis by Hong et al. comparing ESP block, TLIP block, and wound infiltration reported that TLIP resulted in the greatest reduction in opioid consumption and pain scores compared to control, with ESP block providing similar efficacy to TLIP block if performed at the level of the surgical site and wound infiltration presenting an acceptable analgesic alternative when compared to control [6]. The authors hypothesized that the level of injection was more important for the ESP than TLIP and that TLIP is more appropriate for multilevel analgesia due to its greater ease of performance [6]. Khor et al. studied ESP and TLIP blocks in their 2025 pairwise meta-analysis and found no significance between the efficacy of the two blocks in terms of postoperative pain scores and opioid consumption [2]. A systematic review and meta-analysis by Peng et al. by contrast reported that patients who received ESP had lower pain scores and decreased postoperative opioid consumption compared to those who received TLIP and had no significant difference in intraoperative opioid consumption or incidence of adverse events such as nausea, vomiting, postoperative sedation, and respiratory depression [11]. A third systematic review and trial sequential meta-analysis by Majage et al. sought to build on the study by Peng et al. with a larger patient cohort [21]. Their findings reported lower pain scores, lower 24-hour opioid consumption, and no significant difference in opioid-related side effects in patients who received ESP compared to TLIP blocks. The authors posited that the difference could be due to TLIP’s targeting only the sensory dorsal rami of the spinal nerves. Of note, the three meta-analyses comparing ESPs and TLIPs specifically each included 5–6 studies [2, 11, 21], all of which shared at least four of the same RCTs. Studies have been limited by the high heterogeneity, lack of standard block methodologies in these studies, and relatively short follow-up periods (24–48 h).

One study comparing paravertebral, TLIP, and retrolaminar blocks found that TLIP and RLB resulted in lower pain scores, lower rescue opioid requirements, and higher recovery scores than PVB [3]. Incidence of motor blockade was highest in the PVB group, followed by TLIP, then RLB, with no motor blockade in the control group.

Three studies compared the efficacy of classical and modified TLIPs. In a prospective, randomized trial of sixty patients by Ciftci et al., mTLIP was found to have a higher success rate of one-time block and shorter block times but overall similar quality of analgesia as cTLIP as determined by intra- and post-operative opioid consumption, rescue analgesic use, and active/passive VAS scores [23]. The study reported no difference in opioid-related adverse effect profiles or block-related complications. A prospective randomized, single-blinded controlled pilot study on eight patients undergoing posterior lumbar decompression and stabilization surgery by Tantri et al. found that mTLIP resulted in lower IL-6 levels (as a marker of pain-related inflammation) and pain scores at 12 h postoperatively compared to cTLIP but no difference in total postoperative morphine consumption at 24 h between the two groups [26]. A comparative, randomized, parallel assignment trial by Mondal et al. found significantly higher opioid consumption in the first 24 postoperative hours, higher rescue analgesic requirement, higher numeric pain scores, higher PONV rates, and lower time to first analgesic request in patients that received multimodal analgesia without block and comparable outcomes in these measures between the mTLIP and cTLIP groups [22].

## Retrolaminar (RLB) Blocks

### Mechanism

First performed in 2006 by Pfeiffer [27] as an alternative to the paravertebral block for analgesia in a mastectomy patient, the retrolaminar block (also known as the paravertebral lamina technique) provides analgesia by targeting the paraspinal nerves that reside between the deep paraspinal muscles (i.e. superior costotransversospinalis muscle) and the vertebral lamina [7, 28].

### Technique

The RLB can be performed with the patient in the prone or sitting positions, and the technique has been described with both 1–7 MHz convex array and higher-frequency (4–16 MHz, 12–15 MHz) linear-array ultrasound transducers [1,38886235,9]. The ultrasound probe is placed longitudinally in the sagittal orientation to visualize the vertebral laminae (1–2 cm lateral to the spinous process), and a needle is advanced in-plane until contact is made with the posterior surface of the lamina [16–18, 28]. Correct placement of the needle tip is confirmed by visualization of linear spread of the local anesthetic in the fascial plane between the deep paraspinal muscles and the lamina.

### Outcomes

Compared to control, administration of RLB has been shown to improve extubation, recovery, and PACU discharge times, as well as to improve pain scores and time to first rescue analgesic in the recovery area [29]. Although one randomized double blind single center study by Kamel et al. showed paradoxically increased pain scores at 8 h and no significant difference at 12 h and 24 h postoperatively [29], another prospective observational study by Peker et al. found decreased intraoperative anesthetic requirements, decreased postoperative pain medication consumption, and decreased pain scores up to 24 h postoperatively [28]. A single-center prospective randomized control trial by Tas et al. also showed improved pain control at 0–1 h, 2–12 h, and 24 h compared to control following lumbar disc herniation surgery [3]. The effect was observed to be comparable to or more consistent than the analgesia provided by TLIP, and more effective than the analgesia conferred by PVB while maintaining lower incidence of motor blockade.

In a cadaver study by Yang et al., dye injected into the RLB space was demonstrated to spread vertically between the posterior surface of the vertebral laminae and the transversospinalis muscle but was limited laterally by the intertransverse ligaments [17]. In ESP blocks, more lateral spread over the intertransverse ligaments was observed, and more thoracic spinal nerves were stained (median 2.0 and 3.5, respectively). Both techniques resulted in spread to the back muscles posteriorly and into the ipsilateral paravertebral space by means of the superior costotransverse ligament. Similarly, an MRI study by Adhikary et al. demonstrated relatively limited lateral spread of contrast in RLB between the spinous process and the edge of the bony lamina compared to the wider spread into the intercostal spaces (in exchange for less transversospinalis staining) in ESPB [18]. Both ESP and RLB resulted epidural and neural foraminal spread over 2 to 5 levels, although the ESPB also achieved a greater craiocaudal spread.

Clinically, comparisons between RLB and ESPB have demonstrated greater efficacy of the ESPB. In a single center prospective, randomized, double-blind study by Kyenshilik et al., the ESP group had lower numeric pain scores at 12 and 24 h as well as lower total opioid consumption than the RLB group [7]. However, there were no significant differences in pain scores at 30 m, 1 h, or 6 h, and no statistically significant differences in recovery quality score, time to mobilization, or time to discharge.

## Inter-semispinal Fascial Plane Block (ISPB)

### Mechanism

Also known as the cervical cervicis plane (CCeP) block, the inter-semispinal fascial plane block is a modification of the multifidus cervicis plane block and targets the dorsal rami of the cervical spinal nerves in the fascial plane between the semispinalis cervicis and the semispinalis capitis muscles [18, 19, 30–32]. The CCeP is a continuous fascial plane that runs from the nuchal line of the occiput to C7, and the dorsal rami that run through this plane innervate the posterior cervical muscles and the skin of the neck [31, 32].

### Technique

The ISP can be subdivided into upper and lower zones [31]. In the upper zone at the level of C2, the ISP runs between the obliquus capitis inferior muscle and semispinalis capitis muscle [32]. Relevant anatomic structures include the C2 spinous and articular processes, the semispinalis capitis and obliquus capitis inferior muscles, and potentially the occipital nerve [31]. In the lower zone at the level of C5, relevant structures include the C5 spinous process; C5 lamina; and the trapezius, splenius, semispinalis capitis, semispinalis cervicis, and multifidus muscles. The deep cervical arteries and veins may also be visualized in this plane.

The ISPB can be performed in either sedated or awake patients in the prone position [18, 19]. The relevant musculature is identified at the level of the posterior articular process of the desired cervical vertebra [32]. A needle is advanced in-plane in the lateral-to-medial direction until the tip is positioned between the semispinalis capitis and semispinalis cervicis muscles. Proper needle tip placement may be confirmed through hydrodissection [19, 31].

### Outcomes

In this literature search, two randomized control studies examining the efficacy of ISPB in posterior cervical spine surgery were found. One by Abdelhaleem et al. [33] looking at 52 patients in a single center demonstrated decreased total opioid consumption in the intra- and immediate post-operative periods (although this finding may in part be confounded by the limited use of multimodal analgesia per Xue et al. [34] no significant hemodynamic perturbations compared to control [18], and lower numerical pain scores at rest compared to control. An RCT by Ramachandran, et al. showed improved pain control, lower opioid and muscle relaxant use, decreased intraoperative bleeding, shorter surgical times (attributed to lower hemodynamic variability), and earlier mobilization times in the intervention group [19]. A cadaver study by Leurcharusmee et al. demonstrated that dye injected at the C2 and C5 levels consistently stained the dorsal rami [31]. Spread patterns with larger volumes (10 cc at C2 and 20 cc at C5) extended to the contralateral C2 dorsal rami superiorly and to the ipsilateral T2 inferiorly while more conservative volumes (5 cc at C2 and 10 cc at C5) spread about 5 levels each. The injections, however, did not spread beyond the C2 level of the CCeP superiorly, nor did they stain the multifidus muscle, the retrolaminar region, or the ventral rami. This finding was consistent with sparing of the brachial plexus and phrenic nerve as well as MEPs and SSEPs noted in other studies [19, 32]. ISPB has also been observed in a prospective case series to provide effective pain control in the immediate postoperative period for pediatric patients undergoing suboccipital surgery for Chiari decompression when performed at the C3 level bilaterally [35].

## Epidural Analgesia (EA)

### Mechanism

As its name suggests, epidural analgesia seeks to provide pain relief by delivering medication (such as local anesthetic, opioid, or steroids) into the epidural space and targeting the structures that reside there. In a cadaveric study examining the distribution of ink injected into the epidural space, not only the structures of the vertebral canal but also those of the intervertebral foramina were found to be stained [36]. Ink spread circumferentially within the vertebral canal and radially within the intervertebral foramina, bathing the dorsal root ganglia and nerve root sheathes, and often following the spinal nerve beyond the ganglion. The posterior longitudinal ligament acted as a barrier directing the flow of injectate, routing the ink in numerous small channels along the spinal nerve sleeve rather than through fat further away from the nerve.

### Technique

For spine surgery, epidural analgesia may be achieved through a single bolus of anesthetic administered preoperatively by the anesthesiologist or via an infusion catheter placed by the surgeon under direct visualization intraoperatively [5, 12, 37, 38]. In the case of single epidural injection, the anesthesiologist advances an 18 g Touhy needle into the epidural space via the midline or paramedian approach [38]. The patient may be positioned in the lateral or upright position. Following loss of resistance to saline or air, the dose of local anesthetic is administered, and the Touhy needle is withdrawn. For intraoperative catheter placement, the surgeon places one or two epidural catheters under the desired lamina, visualized in the surgical field at the end of the procedure [5, 12, 39]. Following administration of an initial bolus of local anesthetic, the wound may be irrigated and closed and the catheter removed, or the catheter may be tunneled through intact skin and left in place for continuous infusion of local anesthetic.

### Outcomes

Perhaps the technique with the most published recommendations, epidural analgesia has been recommended by several guidelines to varying degrees. In 2021, the European Society of Regional Anaesthesia and Pain therapy (ESRA) published recommendations on pain management after complex spine surgery on behalf of the PROSPECT Working Group [5]. These guidelines recommended the use of “epidural analgesia with local anaesthetics alone or combined with opioids…as a component of multimodal analgesia” but did not recommend epidural opioids alone due to limited evidence. Similarly, the 2021 ERAS Guideline and Consensus Statement for perioperative care in lumbar spinal fusion gave strong recommendations for the use of epidural analgesia in controlling postoperative pain with high quality of evidence [1]. Further recommendations included the use of long-acting local anesthetics (LA) or a combination of epidural opioid and LA over morphine alone, the use of epidural analgesia earlier in the surgical course, and a small dose of LA early on to mitigate the possible transient motor deficit. The only guideline that did not recommend epidural analgesia was the 2021 PROSPECT recommendations on pain control after laminectomy, which cited lack of evidence of clinically relevant pain relief over multimodal analgesia combined with wound infiltration and the risk of adverse effects [14].

More recently, a systematic review, meta-analysis, and TSA of pain management following lumbar discectomy by Zachodnik et al. found a statistically significant reduction in opioid consumption compared with control at 24 postoperative hours in five trials with high risk of study bias and low quality of evidence [4]. Analysis of eight and nine trials that included pain at rest at 6 ± 2 and 24 postoperative hours respectively found statistically significant reduction in NRS compared with control, high risk of bias, and low quality of evidence at both time points. Limitations included assessment of pain predominantly at rest without sufficient attention to other outcomes, lack of transparency regarding baseline or background multimodal analgesic regimens, and the overall low quality of evidence of the included trials.

Perhaps in an effort to address the risk of adverse effects such as loss of sensory function, motor weakness, delayed diagnosis of neurological complications, catheter failure, and the rare but potentially devastating epidural hematomas or vascular puncture [5, 12], some more recent studies have analyzed the efficacy of single epidural boluses rather than continuous catheters. An RCT of 38 patients undergoing elective open laminectomy with fusion by Thepsoparn et al. found decreased length of stay, lower numeric pain scores at rest, lower postoperative opioid consumption at 24 h, higher patient satisfaction, shorter operative time, and decreased intraoperative blood loss in patients who received single dose of epidural bupivacaine plus morphine compared to control [38]. However, the intervention group also showed greater intraoperative hypotension. There was no reported difference in postoperative complications. Another RCT by Hermans et al. of 20 patients undergoing lumbar interlaminar decompressive spine surgery showed a statistically significant decrease in numerical pain scores at up to 48 postoperative hours in patients who received a single dose of intraoperative epidural bupivacaine as compared to control [39]. This difference was most pronounced in the immediate postoperative period. The study also found no significant difference in length of stay, surgical complication rate, total opioid consumption, or patient satisfaction or recovery index measures.

In pediatric literature, a 2019 Cochrane review examining epidural vs. systemic analgesia following thoraco-lumbar spine surgery found that EA resulted in lower pain scores both at rest and with movement at all time points up to 72 postoperative hours [37]. There was some evidence of decreased time to return of bowel function and first PO intake compared to control, and no difference in nausea and vomiting. The review was unable to determine difference in time to ambulation; length of hospital stay; or risk of complications such as respiratory depression, wound infection, epidural abscess, or neurological complications; and was unable to comment on the safety of the technique due to the very low quality of evidence.

## Intrathecal Administration of Opioids

### Mechanism

Intrathecal opioids provide pain relief by acting at the level of the dorsal horn both pre- and post-synaptically [40]. Pre-synaptically, excitatory neurotransmitter (glutamate and substance P) release is decreased through adenylate cyclase inhibition, decreased calcium ion entry, and decreased intracellular calcium levels. Post-synaptically, increased efflux of potassium ions hyperpolarizes the membrane. As a hydrophilic molecule, morphine is cleared less effectively by the capillaries of the spinal cord than other opioids, and its effects may last up to 24 h [8, 10]. The inhibitory neurotransmitters glycine and GABA may also play a role by activating descending inhibitory pathways.

### Technique

The intrathecal space is accessed under aseptic conditions via lumbar puncture using a spinal needle (often 25G Quincke) in the lateral or upright position [8]. After confirming intrathecal placement with return of CSF, a bolus of preservative-free morphine is administered. A wide range of morphine dosing have been reported – from 100mcg to 1 mg – but 300mcg has been recommended previously, perhaps due to the ceiling effect noted at doses > 500mcg in drug efficacy and incidence of adverse effects [10].

### Outcomes

Although ITM has been used since 1979 for analgesic purposes [10], recommendations for it use for postoperative pain following spine surgery have been mixed. The 2021 ERAS Guideline and Consensus Statement for perioperative care in lumbar spinal fusion gave strong recommendations for the use of ITM in controlling postoperative pain with high quality of evidence, citing reduced postoperative pain scores and decreased postoperative opioid consumption without risk of significant adverse effects (although the incidence of pruritus may be increased and be addressed with naloxone) [1]. However, neither the 2021 PROSPECT recommendations for pain management following complex spine surgery nor the 2021 PROSPECT recommendations for pain management following laminectomy recommend the use of ITM, the former citing lack of evidence and the latter due to the risk of adverse effects, especially in the outpatient setting [5, 14]. A meta-analysis and systematic review by Wang et al. also from 2021 looking at the efficacy and safety of ITM for patients undergoing spine (mostly lumbar) surgery found statistically significant reductions in pain scores at several time points in the first 24 h postoperatively (up to ~ 2 points on a 10 cm Visual Analog Scale), with the greatest effect observed at 4 h, and a taper in the analgesic benefit to no significant difference at 48 h in patients who received ITM [10]. When compared to the findings of other meta-analyses, the authors found this effect to be greater than those seen with ketamine supplementation or wound infiltration with local anesthetic. Wang et al.’s work also indicated reduced analgesic consumption in the ITM group (15.59 mg morphine spared in the first 24 h) but no statistically significant difference in length of hospital stay or incidence of adverse events such as nausea, vomiting, sedation, respiratory depression (incidence 1.8%, pooled relative risk 3.27), headache, or urinary retention as compared to control. The incidence of pruritus, however, was significantly increased in the ITM group (20-59.5%). The time to first analgesic request could not be analyzed due to insufficient data. The certainty of evidence was determined to be “moderate,” and the study was limited by significant heterogeneity in all meta-analyses except pooled analysis of adverse events. More recently, a 2023 double-blind prospective comparative study by Mahmoud et al. comparing ESPB and ITM found that while ITM 300mcg provided more potent analgesia up to 48 h postoperatively by VAS scores (higher in the ESP block group at all time points), time to first analgesic request, and postoperative analgesic requirements, the increased analgesic effect was also associated with decreased postoperative oxygen saturation at all time points as well as increased urinary retention (~ 30% of cases) and sedation scores, although the last did not result in need for intervention [8]. A narrative review by Reysner et al. found nine studies related to regional anesthesia techniques in the management of pain following scoliosis surgery and recommended the use of more peripheral nerve blocks like ESPB due to its greater safety profile as compared to ITM with the latter’s higher incidence of respiratory depression, pruritus, nausea, and vomiting [12]. A 2025 systematic review of pain management following lumbar discectomy with meta-analysis and TSA by Zachodnik et al. found two trials examining cumulative opioid consumptions at 24 postoperative hours with significant decrease in opioid consumption with ITM compared to control, high risk of bias, and low quality of evidence. Five trials reported on pain at rest after 6 + 2 h postoperatively with statistically significant reduction in NRS compared to control, high risk of bias, and very low quality of evidence [4]. Limitations of the study included assessment of pain predominantly at rest without sufficient attention to other outcomes, lack of transparency regarding baseline or background multimodal analgesic regimens, and the overall low quality of evidence of the included trials.

## Potential Effects of Regional Anesthesia on Intraoperative Neuromonitoring

An important consideration when incorporating regional anesthesia into spine surgery is its potential influence on intraoperative neurophysiological monitoring (IONM), particularly somatosensory evoked potentials (SSEPs) and motor evoked potentials (MEPs) [41]. Neuraxial techniques, including epidural and spinal anesthesia, may attenuate or abolish evoked responses through local anesthetic blockade of sensory and motor conduction pathways, potentially complicating the interpretation of intraoperative neurological changes [12]. In contrast, deep fascial plane blocks primarily target peripheral sensory pathways and are generally less likely to interfere with IONM when local anesthetic spread remains confined to the intended tissue planes. However, the relatively large volumes of local anesthetic frequently administered with these techniques may result in unintended spread into the paravertebral or epidural spaces [42], potentially affecting spinal nerve roots or neural conduction and confounding SSEP or MEP interpretation [41]. The magnitude of this effect likely depends on the block location, injection volume, local anesthetic concentration, extent of spread, and timing relative to baseline IONM acquisition. Although direct evidence demonstrating clinically significant IONM interference from ESPB or TLIP blocks during spine surgery remains limited [12, 43], this potential interaction warrants consideration, particularly in procedures requiring reliable multimodal neuromonitoring. Coordination among the anesthesiology, surgical, and neurophysiology teams is therefore essential, with consideration given to obtaining baseline signals before regional anesthetic administration when feasible, using the lowest effective local anesthetic volume, and distinguishing block-related signal changes from possible surgical neurological injury.

## Comparative Safety Profiles of Regional Anesthetic Techniques in Spine Surgery

The selection of regional anesthetic techniques for spine surgery requires careful consideration of their relative benefits and potential complications, particularly in patients undergoing procedures where postoperative neurological assessment is essential. Neuraxial techniques, including epidural and spinal anesthesia, can provide effective analgesia but carry risks of motor blockade, hypotension, and rare but potentially catastrophic complications such as epidural hematoma and direct neurological injury. Motor blockade following neuraxial local anesthetic administration may delay recognition of new postoperative neurological deficits or confound the interpretation of deficits related to surgical nerve injury. In contrast, posterior fascial plane blocks primarily target posterior sensory pathways and are generally less likely to produce clinically significant motor blockade. Nevertheless, unintended spread of local anesthetic into the paravertebral or epidural spaces may theoretically result in motor weakness, particularly with high-volume injections. Local anesthetic systemic toxicity remains a potential complication of all local anesthetic techniques, especially with fascial plane blocks using large volumes of medication combined with surgical local infiltration [44]. Although posterior fascial plane blocks avoid intentional needle placement within the neuraxial space, bleeding and hematoma formation remain possible, particularly in patients receiving anticoagulant or antiplatelet therapy. While epidural hematoma is a recognized complication of neuraxial procedures, the risk associated with ESPB, TLIP, and RLB is less well characterized [45], and the absence of minimal reported complications should not be interpreted as evidence of equivalent safety across techniques. A comparative summary of these considerations is provided in Table 2.

**Table 2:** Comparative safety profiles of regional anesthetic techniques in spine surgery

Technique	Motor blockade / masking neurological injury	LAST risk	Hematoma / bleeding risk	Principal advantages
Epidural analgesia	High; local anesthetic may produce motor weakness and obscure postoperative deficits	Possible, particularly with inadvertent intravascular injection or excessive dosing	Rare but potentially catastrophic epidural hematoma	Dense, titratable analgesia; catheter-based delivery
Spinal anesthesia	High during active blockade; may delay neurological assessment	Possible, but systemic toxicity is uncommon with typical intrathecal doses	Rare but potentially catastrophic neuraxial hematoma	Reliable anesthesia with relatively small local anesthetic doses
ESPB	Generally low; unintended paravertebral or epidural spread may cause weakness	Relevant concern with bilateral, high-volume injections	Bleeding possible; risk of clinically significant hematoma incompletely characterized	Broad multilevel analgesic coverage
TLIP block	Generally low; unintended spread or direct nerve involvement may cause weakness	Relevant concern with bilateral, high-volume injections	Bleeding possible; risk of clinically significant hematoma incompletely characterized	Targeted posterior analgesia
RLB	Generally low, but deeper spread may produce motor effects	Relevant concern with bilateral, high-volume injections	Bleeding possible; risk of clinically significant hematoma incompletely characterized	Targeted posterior posterior analgesia

Abbreviations: *ESPB* erector spinae plane block, *LAST *local anesthesia systemic toxicity, *RLB* retrolaminar block, *TLIP* thoracolumbar interfascial plane

## Practical Considerations for Regional Anesthesia in the Prone Position

The performance of regional anesthetic techniques in patients undergoing spine surgery presents several practical challenges related to patient positioning, procedural timing, and technical feasibility. Although ESPB, TLIP, and RLB can be performed in the prone position, their integration into the perioperative workflow requires careful coordination. Performing these blocks preoperatively in awake patients may allow assessment of neurological function and block-related complications before induction of general anesthesia; however, positioning patients with severe back pain, spinal instability, or neurological deficits may not be feasible. Alternatively, blocks may be performed after induction and prone positioning in the operating room, which would eliminate the need for additional repositioning but potentially prolong operating room time and precluding immediate patient-reported assessment of neurological symptoms. Whenever possible, block placement should be planned to minimize operating room delays.

## Age- and Population-Specific Considerations

The efficacy and safety of regional anesthetic techniques in spine surgery may differ substantially between adult patients undergoing degenerative spine procedures and pediatric patients undergoing scoliosis correction or Chiari decompression [46]. In adults, ESPB, TLIP, and retrolaminar blocks have been investigated primarily in lumbar decompression and fusion procedures, with studies suggesting reductions in early postoperative pain scores, opioid consumption, and rescue analgesic requirements, although findings vary by technique, procedure, and study design. In pediatric populations, particularly adolescents undergoing posterior spinal fusion for scoliosis, ESPB has demonstrated potential opioid-sparing and analgesic benefits [46]; however, the available evidence is less extensive, and differences in analgesic protocols limit direct extrapolation from adult studies. Local anesthetic dosing represents an important distinction, as pediatric patients require stricter weight-based calculations, particularly when bilateral or multilevel fascial plane blocks are performed. Although adult dosing is also constrained by patient weight, comorbidities, and cumulative local anesthetic exposure, the relatively smaller body mass of pediatric patients increases the importance of accounting for total administered dose. Furthermore, age-dependent pharmacokinetic differences, particularly in infants and younger children, may influence systemic local anesthetic exposure and susceptibility to toxicity [47]. Although posterior fascial plane blocks generally have a low likelihood of clinically significant motor blockade, unintended neuraxial spread remains a concern in both populations, particularly with high-volume injections [42]. Importantly, no universally validated local anesthetic volume or concentration threshold has been established for ESPB, TLIP, or retrolaminar blocks that guarantees preservation of motor or sensory evoked potentials. Accordingly, regional anesthetic techniques should be individualized according to patient age, body weight, surgical procedure, anatomical considerations, and monitoring requirements, with careful attention to cumulative local anesthetic dosing and postoperative neurological evaluation.

## Economic and Operational Considerations

Beyond improvements in postoperative analgesia and reductions in opioid consumption, the economic and operational implications of incorporating regional anesthesia into spine surgery pathways warrant consideration. Although these fascial plane blocks may reduce postoperative analgesic requirements and facilitate early recovery, evidence directly evaluating their cost-effectiveness, operating room efficiency, and resource utilization in spine surgery remains limited. Implementation of regional anesthesia protocols for spine surgery requires additional resources, including trained regional anesthesia personnel, ultrasound equipment, sterile supplies, local anesthetic medications, and potentially a dedicated time for block placement and monitoring [48]. The effect on perioperative workflow depends substantially on when and where the block is performed. Blocks performed in a dedicated preoperative area or in parallel with other perioperative activities may minimize disruption to operating room schedules [49], whereas blocks performed after induction of general anesthesia may increase anesthesia-controlled time and delay surgical incision. Potential downstream economic benefits include reduced rescue analgesic administration, fewer opioid-related adverse effects, decreased postanesthesia care unit utilization, and earlier mobilization or hospital discharge; however, these benefits have not been consistently translated into demonstrated cost savings in spine-specific economic analyses. Furthermore, reductions in postoperative pain or opioid consumption do not necessarily translate into shorter operating room turnover times, which are influenced by numerous institutional and logistical factors. The cost-effectiveness of regional anesthesia is therefore likely to depend on institutional block workflows, procedural volume, staffing models, surgical complexity, and the magnitude of downstream recovery benefits. Notably, a recent retrospective cost analysis of approximately 600 patients undergoing lumbar fusion found that regional plane blocks were associated with reduced pain during activity and shorter postanesthesia care unit stays but also longer surgical times and higher adjusted hospital billed costs compared with no block [50]. These findings underscore that improvements in analgesic outcomes do not necessarily translate into greater perioperative efficiency or reduced healthcare expenditures, and that the economic value of regional anesthesia should be evaluated within the context of the overall surgical pathway. Future prospective studies should incorporate formal economic evaluations and operational outcomes, including block performance time, anesthesia-controlled time, operating room turnover, postanesthesia care unit length of stay, total hospitalization costs, and resource utilization, to determine whether routine implementation provides measurable value beyond improvements in conventional analgesic outcomes.

## Conclusion

Taken together, the available evidence suggests that regional and neuraxial techniques can improve analgesia following spine surgery, although several important limitations preclude strong conclusions regarding the superiority of any individual technique. Among outcomes evaluated in large meta-analyses, reduction in postoperative nausea and vomiting appears to be one of the most consistent findings, supported by high-certainty evidence with little between-study heterogeneity. In contrast, estimates of analgesic efficacy and opioid reduction are frequently limited by substantial heterogeneity and a lack of standardized block techniques, including differences in local anesthetic selection, volume, concentration, timing, and level of injection. Importantly, analgesic efficacy may depend as much on selecting an appropriate block level for the surgical site as on the specific fascial plane block performed. Neuraxial techniques may provide more profound and prolonged analgesia but must be balanced against their potential for more consequential adverse effects. Given the absence of compelling evidence favoring a single regional technique across procedures, clinicians may derive greater benefit from developing proficiency in a limited repertoire of blocks and applying them at anatomically appropriate levels rather than selecting among numerous techniques based primarily on reported procedure-specific associations. This approach prioritizes technical proficiency, anatomic rationale, and appropriate block selection while the comparative evidence continues to evolve.

## Key References

- Debono B, Wainwright TW, Wang MY, Sigmundsson FG, Yang MMH, Smid-Nanninga H, Bonnal A, Le Huec JC, Fawcett WJ, Ljungqvist O, Lonjon G, de Boer HD. Consensus statement for perioperative care in lumbar spinal fusion: Enhanced Recovery After Surgery (ERAS(R)) Society recommendations. Spine J 2021;21:729-52.
  - ○ Provides a consensus statement of overall perioperative care for lumbar spine surgery. Under the direction of the ERAS society, the manuscript provides a multidisciplinary guideline from international experts.
- Waelkens P, Alsabbagh E, Sauter A, Joshi GP, Beloeil H, Anaesthesia PWgotESoR, Pain t. Pain management after complex spine surgery: A systematic review and procedure-specific postoperative pain management recommendations. Eur J Anaesthesiol 2021;38:985-94.
  - ○ Provides a review for pain management for complex spine surgery that aimed to evaluate the available literature and develop recommendations for optimal pain management after complex spine surgery.
- Dost B, Turunc E, Karapinar YE, Beldagli M, Turan EI, Dokmeci H, De Cassai A. Erector spinae plane block for postoperative analgesia in vertebral surgery: An updated meta-analysis of randomized controlled trials with trial sequential analysis and meta-regression. J Clin Anesth 2026;111:112184.
  - ○ A meta-analysis of 60 trials that evaluates the analgesic efficacy of the erector spinae plane block in adults undergoing spine surgery. The review paper aims to determine whether the available evidence is sufficient to support definitive conclusions.

## Data Availability

No datasets were generated or analysed during the current study.

## References

[1] Debono B, Wainwright TW, Wang MY, Sigmundsson FG, Yang MMH, Smid-Nanninga H, Bonnal A, Le Huec JC, Fawcett WJ, Ljungqvist O, Lonjon G, de Boer HD. Consensus statement for perioperative care in lumbar spinal fusion: Enhanced Recovery After Surgery (ERAS(R)) Society recommendations. Spine J. 2021;21:729–52. 10.1016/j.spinee.2021.01.001 33444664

[2] Khor WT, Chang Y, Tien CH, Chen LY, Hsu HH, Perng PS, Wong CE, Hsu HJ, Lee JS. Erector spinae plane block versus thoracolumbar interfascial plane block in lumbar spine surgery: a meta-analysis of randomized controlled trials. Global Spine J. 2025;15:1367–74. 10.1177/21925682241266518 PMC11559919 38914010

[3] Tas HG, Akyol F, Somunkiran M. Comparison of paravertebral, thoracolumbar interfascial plane, and retrolaminar blocks for postoperative analgesia in lumbar disc herniation surgery: A randomized controlled trial. Med (Baltim). 2026;105:e47141. 10.1097/MD.0000000000047141 PMC12794954 41517760

[4] Zachodnik J, Bech-Azeddine R, Sandberg M, Scherwin R, Hartvigsen Gronholm Jepsen RM, Jorgensen LM, Thybo KH, Geisler A. Postoperative pain management after lumbar discectomy. A Systematic Review With Meta-Analyses and Trial Sequential Analyses. Eur J Pain. 2026;30:e70261. 10.1002/ejp.70261 PMC13067818 41960853

[5] Waelkens P, Alsabbagh E, Sauter A, Joshi GP, Beloeil H, Anaesthesia, PWgotESoR. Pain t. Pain management after complex spine surgery: A systematic review and procedure-specific postoperative pain management recommendations. Eur J Anaesthesiol. 2021;38:985–94. 10.1097/EJA.0000000000001448 PMC8373453 34397527

[6] Hong B, Baek S, Kang H, Oh C, Jo Y, Lee S, Park S. Regional analgesia techniques for lumbar spine surgery: a frequentist network meta-analysis. Int J Surg. 2023;109:1728–41. 10.1097/JS9.0000000000000270 PMC10389589 36912781

[7] Kyenshilik D, Sari S, Gulasti F, Umutlu Y. Comparison of the effects of retrolaminar block (RLB) and erector spinae plane (ESP) block on postoperative recovery quality and pain in lumbar spinal surgery. Eur Spine J. 2026;35:2051–7. 10.1007/s00586-025-09273-2 40911071

[8] Mahmoud AM, Ragab SG, Shawky MA, Masry DH, Botros JM. The efficacy of erector spinae plane block compared with intrathecal morphine in postoperative analgesia in patients undergoing lumbar spine surgery: a double-blind prospective comparative study. Pain Physician. 2023;26:149–59. 36988361

[9] Patel TD, McNicholas MN, Paschell PA, Arnold PM, Lee CT. Thoracolumbar Interfascial Plane (TLIP) block verses other paraspinal fascial plane blocks and local infiltration for enhanced pain control after spine surgery: a systematic review. BMC Anesthesiol. 2024;24:122. 10.1186/s12871-024-02500-1 PMC10976846 38539065

[10] Wang J, Sun H, Sun WT, Sun HP, Tian T, Sun J. Efficacy and safety of intrathecal morphine for pain control after spinal surgery: a systematic review and meta-analysis. Eur Rev Med Pharmacol Sci. 2021;25:2674–84. 10.26355/eurrev_202103_25431 33829454

[11] Peng Q, Meng B, Yang S, Ban Z, Zhang Y, Hu M, Zhao W, Wu H, Tao Y, Zhang L. Efficacy and safety of erector spinae plane block versus thoracolumbar interfascial plane block in patients undergoing spine surgery: a systematic review and meta-analysis. Clin J Pain. 2024;40:114–23. 10.1097/AJP.0000000000001177 37982694

[12] Reysner M, Kowalski G, Geisler-Wojciechowska A, Resyner T, Wieczorowska-Tobis K. Anesthesia and pain management for scoliosis surgery: a narrative review. Clin Spine Surg. 2025;38:259–65. 10.1097/BSD.0000000000001758 PMC12180691 39749911

[13] Long G, Liu C, Liang T, Zhan X. The efficacy of thoracolumbar interfascial plane block for lumbar spinal surgeries: a systematic review and meta-analysis. J Orthop Surg Res. 2023;18:318. 10.1186/s13018-023-03798-2 PMC10127357 37095532

[14] Peene L, Le Cacheux P, Sauter AR, Joshi GP, Beloeil H, Collaborators PWG. European Society of Regional A. Pain management after laminectomy: a systematic review and procedure-specific post-operative pain management (prospect) recommendations. Eur Spine J. 2021;30:2925–35. 10.1007/s00586-020-06661-8 33247353

[15] Forero M, Adhikary SD, Lopez H, Tsui C, Chin KJ. The erector spinae plane block: a novel analgesic technique in thoracic neuropathic pain. Reg Anesth Pain Med. 2016;41:621–7. 10.1097/AAP.0000000000000451 27501016

[16] Onishi E, Toda N, Kameyama Y, Yamauchi M. Comparison of clinical efficacy and anatomical investigation between retrolaminar block and erector spinae plane block. Biomed Res Int. 2019;2019:2578396. 10.1155/2019/2578396 PMC6458933 31032339

[17] Yang HM, Choi YJ, Kwon HJ, Cho OJ, Kim TH. Comparison of injectate spread and nerve involvement between retrolaminar and erector spinae plane blocks in the thoracic region: a cadaveric study. Anaesthesia. 2018;73:1244–50. 10.1111/anae.14408 30113699

[18] Adhikary SD, Bernard S, Lopez H, Chin KJ. Erector spinae plane block versus retrolaminar block: a magnetic resonance imaging and anatomical study. Reg Anesth Pain Med. 2018;43:756–62. 10.1097/AAP.0000000000000798 29794943

[19] Ramachandran K, Chandramohan M, Shetty AP, Subramanian B, Kanna RM, Rajasekaran S. Efficacy and safety of ultrasound guided inter-semispinal plane block for postoperative analgesia in posterior cervical laminectomy - a prospective randomised controlled study. Global Spine J. 2025;15:1625–34. 10.1177/21925682241254327 PMC11571355 38728581

[20] Dost B, Turunc E, Karapinar YE, Beldagli M, Turan EI, Dokmeci H, De Cassai A. Erector spinae plane block for postoperative analgesia in vertebral surgery: An updated meta-analysis of randomized controlled trials with trial sequential analysis and meta-regression. J Clin Anesth. 2026;111:112184. 10.1016/j.jclinane.2026.112184 41861494

[21] Majage S, Ravikumar RH, Prasanna M, Chandramouli M, Datta PK, Baidya DK. Comparison of efficacy of ultrasound-guided erector spinae plane block versus thoracolumbar interfascial plane block in patients undergoing lumbar spine surgeries: A systematic review and trial sequential meta-analysis. Indian J Anaesth. 2024;68:752–61. 10.4103/ija.ija_373_24 PMC11460799 39386407

[22] Mondal S, Pandey RK, Kumar M, Sharma A, Darlong V, Punj J. Analgesic efficacy of classical thoracolumbar interfascial plane block versus modified thoracolumbar interfascial plane block in patients undergoing lumbar disc surgeries: A comparative, randomised controlled trial. Indian J Anaesth. 2024;68:366–73. 10.4103/ija.ija_1153_23 PMC10993931 38586260

[23] Ciftci B, Ekinci M. A prospective and randomized trial comparing modified and classical techniques of ultrasound-guided thoracolumbar interfascial plane block. Agri. 2020;32:186–92. 10.14744/agri.2020.72325 33398870

[24] Ahiskalioglu A, Alici HA, Selvitopi K, Yayik AM. Ultrasonography-guided modified thoracolumbar interfascial plane block: a new approach. Can J Anaesth. 2017;64:775–6. 10.1007/s12630-017-0851-y 28243853

[25] Hand WR, Taylor JM, Harvey NR, Epperson TI, Gunselman RJ, Bolin ED, Whiteley J. Thoracolumbar interfascial plane (TLIP) block: a pilot study in volunteers. Can J Anaesth. 2015;62:1196–200. 10.1007/s12630-015-0431-y 26149600

[26] Tantri AR, Sukmono RB, Lumban Tobing SDA, Natali C. Comparing the effect of classical and modified thoracolumbar interfascial plane block on postoperative pain and IL-6 level in posterior lumbar decompression and stabilization surgery. Anesth Pain Med. 2022;12:e122174. 10.5812/aapm-122174 PMC9364521 36061531

[27] Pfeiffer G, Oppitz N, Schone S, Richter-Heine I, Hohne M, Koltermann C. [Analgesia of the axilla using a paravertebral catheter in the lamina technique]. Anaesthesist. 2006;55:423–7. 10.1007/s00101-005-0969-0 16404582

[28] Peker K, Aydin G, Gencay I, Saracoglu AG, Sahin AT, Ogden M, Peker SA. The effect of preemptive retrolaminar block on lumbar spinal decompression surgery. Eur Spine J. 2024;33:4253–61. 10.1007/s00586-024-08219-4 38886235

[29] Kamel AAF, Fahmy AM, Medhat MM, Ali Elmesallamy WAE, Salem DAE. Retrolaminar block for opioid-free anaesthesia and enhanced recovery after posterior lumbar discectomy: A randomised controlled study. Indian J Anaesth. 2024;68:261–6. 10.4103/ija.ija_773_23 PMC10926343 38476544

[30] Adamczyk K, Koszela K, Zaczynski A, Niedzwiecki M, Brzozowska-Mankowska S, Gasik R. Ultrasound-guided blocks for spine surgery: Part 1-Cervix. Int J Environ Res Public Health 2023;20. 10.3390/ijerph20032098 PMC9915556 36767465

[31] Leurcharusmee P, Thunyacharoen S, Inchai C, Mahakkanukrauh P, Shabsigh M, Xu JL. Ultrasound-guided cervical cervicis plane (CCeP) block: a cadaver study. Reg Anesth Pain Med; 2025. 10.1136/rapm-2025-106962 40789719

[32] Xu JL, Shabsigh M. Ultrasound-guided cervical cervicis plane block for postoperative pain control in cervical spine surgery: a technical note. World Neurosurg. 2023;171:5–7. 10.1016/j.wneu.2022.12.044 36528316

[33] Abdelhaleem NF, Youssef EM, Hegab AS. Analgesic efficacy of inter-semispinal fascial plane block in patients undergoing cervical spine surgery through posterior approach: a randomized controlled trial. Anaesth Crit Care Pain Med. 2023;42:101213. 10.1016/j.accpm.2023.101213 36894055

[34] Xue FS, He N, Cheng Y. Assessing analgesic efficacy of regional fascial plane block for posterior cervical spine surgery. Anaesth Crit Care Pain Med. 2024;43:101322. 10.1016/j.accpm.2023.101322 37931830

[35] Pisapia JM, Doherty TM, Grosinger L, Huang A, Muh CR, Abramowicz AE, Xu JL. Safety and preliminary efficacy of cervical paraspinal interfascial plane block for postoperative pain after pediatric chiari decompression. Healthcare (Basel) 2024;12(14):1426. 10.3390/healthcare12141426 PMC11275953 39057568

[36] Hogan Q. Distribution of solution in the epidural space: examination by cryomicrotome section. Reg Anesth Pain Med. 2002;27:150–6. 10.1053/rapm.2002.29748 11915061

[37] Guay J, Suresh S, Kopp S, Johnson RL. Postoperative epidural analgesia versus systemic analgesia for thoraco-lumbar spine surgery in children. Cochrane Database Syst Rev. 2019;1:CD012819. 10.1002/14651858.CD012819.pub2 PMC6360928 30650189

[38] Thepsoparn M, Punyawattanakit P, Jaruwangsanti N, Singhatanadgige W, Chalermkitpanit P. Effects of general anesthesia with and without thoracic epidural block on length of stay after open spine surgery: a single-blinded randomized controlled trial. Spine J. 2022;22:1694–9. 10.1016/j.spinee.2022.05.015 35671941

[39] Hermans SMM, Lantinga-Zee AAG, Droeghaag R, van Santbrink H, van Hemert WLW, Reinders MK, Hoofwijk DMN, van Kuijk SMJ, Rijkers K, Curfs I. A randomized controlled trial using epidural analgesia for pain relief after lumbar interlaminar decompressive spine surgery: the RAPID trial. Spine (Phila Pa 1976). 2024;49:456–62. 10.1097/BRS.0000000000004921 38214681

[40] Rathmell JP, Lair TR, Nauman B. The role of intrathecal drugs in the treatment of acute pain. Anesth Analg. 2005;101:S30–43. 10.1213/01.ANE.0000177101.99398.22 16334491

[41] Pan SJ, Vorhies JS, Lee LH, Lopez JR, Tsui BCH. The effect of intraoperative lidocaine administration in a disrupted erector spinae fascial plane on intercostal transcranial motor evoked potentials. J Clin Anesth. 2022;82:110956. 10.1016/j.jclinane.2022.110956 36029703

[42] Amoroso K, Hughes AP, Sama AA, Cammisa FP, Shue J, Soffin EM. Continuous erector spinae plane catheters leading to unwanted neuraxial spread after spinal fusion surgery: a report of two cases from a terminated prospective randomized clinical trial. Reg Anesth Pain Med. 2023;48:478–81. 10.1136/rapm-2023-104587 37192785

[43] Fujimoto K, Funaba M, Suzuki H, Sakamoto S, Tanaka I, Nishida N, Imajo Y, Yamamoto M, Sakai T. Intraoperative neuromonitoring findings after erector spinae plane block during posterior thoracolumbar fusion surgery. Eur J Orthop Surg Traumatol 2026;36(1):426. 10.1007/s00590-026-05004-5 42944931

[44] Bailey JG, Barry G, Volk T. Local anesthetic dosing for fascial plane blocks to avoid systemic toxicity: a narrative review. Can J Anaesth. 2025;72:1423–47. 10.1007/s12630-025-03034-x 40954415

[45] White LD, Riley B, Davis K, Thang C, Mitchell A, Abi-Fares C, Basson W, Anstey C. Safety of continuous erector spinae catheters in chest trauma: a retrospective cohort study. Anesth Analg. 2021;133:1296–302. 10.1213/ANE.0000000000005730 34473654

[46] Ren Y, Gao J, Nie X, Hua L, Zheng T, Guo D, Zhang J. Bilateral ultrasound-guided erector spinae plane block for postoperative analgesia in paediatric idiopathic scoliosis patients undergoing posterior spine fusion surgery: a randomized controlled trial. Eur Spine J. 2024;33:3823–32. 10.1007/s00586-024-08457-6 39174796

[47] Dontukurthy S, Tobias JD. Update on local anesthetic toxicity, prevention and treatment during regional anesthesia in infants and children. J Pediatr Pharmacol Ther. 2021;26:445–54. 10.5863/1551-6776-26.5.445 PMC8244955 34239395

[48] El-Boghdadly K, Nair G, Pawa A, Onwochei DN. Impact of parallel processing of regional anesthesia with block rooms on resource utilization and clinical outcomes: a systematic review and meta-analysis. Reg Anesth Pain Med. 2020;45:720–6. 10.1136/rapm-2020-101397 32699101

[49] Mazda Y, Peacock S, Wolfstadt J, Matelski J, Chan V, Gleicher YJ. Developing a business case for a regional anesthesia block room: up with efficiency, down with costs. Reg Anesth Pain Med. 2021;46:986–91. 10.1136/rapm-2021-102545 33980698

[50] Solomito MJ, Makanji H. Phila Pa. A cost-benefit analysis of common regional anesthesia blocks used for patients undergoing elective single- or two-level lumbar fusions. Spine (1976) 2026. 10.1097/BRS.0000000000005808 42525721

